# Distribution of the Most Prevalent *Spa* Types among Clinical Isolates of Methicillin-Resistant and -Susceptible *Staphylococcus aureus* around the World: A Review

**DOI:** 10.3389/fmicb.2018.00163

**Published:** 2018-02-12

**Authors:** Parisa Asadollahi, Narges Nodeh Farahani, Mehdi Mirzaii, Seyed Sajjad Khoramrooz, Alex van Belkum, Khairollah Asadollahi, Masoud Dadashi, Davood Darban-Sarokhalil

**Affiliations:** ^1^Department of Microbiology, Faculty of Medicine, Iran University of Medical Sciences, Tehran, Iran; ^2^Department of Microbiology, School of Medicine, Shahroud University of Medical Sciences, Shahroud, Iran; ^3^Department of Microbiology, Faculty of Medicine, Cellular and Molecular Research Center, Yasuj University of Medical Sciences, Yasuj, Iran; ^4^Data Analytics Unit, bioMérieux 3, La Balme Les Grottes, France; ^5^Department of Social Medicine, Faculty of Medicine, Ilam University of Medical Sciences, Ilam, Iran; ^6^Faculty of Medicine, Biotechnology and Medicinal Plants Researches Center, Ilam University of Medical Sciences, Ilam, Iran; ^7^Department of Microbiology, Faculty of Medicine, Shahid Beheshti University of Medical Sciences, Tehran, Iran

**Keywords:** *Staphylococcus aureus*, *spa* typing, MRSA, prevalent, SCC*mec* typing, MLST, clonal complex

## Abstract

**Background:** *Staphylococcus aureus*, a leading cause of community-acquired and nosocomial infections, remains a major health problem worldwide. Molecular typing methods, such as *spa* typing, are vital for the control and, when typing can be made more timely, prevention of *S. aureus* spread around healthcare settings. The current study aims to review the literature to report the most common clinical *spa* types around the world, which is important for epidemiological surveys and nosocomial infection control policies.

**Methods:** A search via PubMed, Google Scholar, Web of Science, Embase, the Cochrane library, and Scopus was conducted for original articles reporting the most prevalent *spa* types among *S. aureus* isolates. The search terms were “*Staphylococcus aureus, spa typing*.”

**Results:** The most prevalent *spa* types were t032, t008 and t002 in Europe; t037 and t002 in Asia; t008, t002, and t242 in America; t037, t084, and t064 in Africa; and t020 in Australia. In Europe, all the isolates related to *spa* type t032 were MRSA. In addition, *spa* type t037 in Africa and t037and t437 in Australia also consisted exclusively of MRSA isolates. Given the fact that more than 95% of the papers we studied originated in the past decade there was no option to study the dynamics of regional clone emergence.

**Conclusion:** This review documents the presence of the most prevalent *spa* types in countries, continents and worldwide and shows big local differences in clonal distribution.

## Introduction

*Staphylococcus aureus*, a leading cause of community-acquired and nosocomial infections, remains a major health problem around the world causing a variety of different conditions including wound infections, osteomyelitis, food poisoning, endocarditis, as well as more life-threatening diseases, such as pneumonia and bacteremia (Goudarzi et al., 2016b). Since the introduction of penicillin into medical therapy in the early 1940s, resistance against beta-lactams started to develop among staphylococcal isolates. To overcome this problem, a narrow spectrum semi-synthetic penicillin (methicillin) was introduced. However, soon after its first use in 1961, the first methicillin-resistant *S. aureus* (MRSA) strain was identified (Turlej et al., 2011). Methicillin resistance is caused by the *mecA* gene product, a modified form of penicillin binding protein (PBP), called PBP2a or PBP2', which has a lower affinity for all beta-lactam antibiotics (Hanssen and Ericson Sollid, 2006). The *mecA* gene is located within the *mec* operon of the staphylococcal cassette chromosome *mec* (SCC*mec*). SCC*mec* typing, which classifies SCC*mec* elements on the basis of their structural differences, is applied in several epidemiological studies of MRSA strains (Turlej et al., 2011). Molecular characterization of *S .aureus* is vital for the rapid identification of prevalent strains and will contribute to the control and prevention of *S. aureus* spread around healthcare settings if results are provided in real time (Siegel et al., 2007; Bosch et al., 2015; O'Hara et al., 2016). Phage typing was originally used for the formal typing of *S. aureus* isolates, but it was gradually replaced by pulsed-field gel electrophoresis (PFGE), the most recent gold standard method for the typing of *S. aureus* isolates (Bannerman et al., 1995; Murchan et al., 2003; Bosch et al., 2015). However, due to its laborious character and difficulties in exchanging data between laboratories, and the requirement for inter-laboratory standardization, PFGE was replaced by multi-locus sequence typing (MLST) and staphylococcal protein A (*spa*) typing (Harmsen et al., 2003). MLST is a great tool for evolutionary investigations and differentiates isolates according to nucleotide variations in 7 housekeeping genes. *Spa* typing, which relies only on the assessment of the number of and sequence variation in repeats at the x region of the *spa* gene, exhibits excellent discriminatory power and has become a useful typing tool for the sake of its ease of performance, cheaper procedure, and standardized nomenclature (Frenay et al., 1996; Koreen et al., 2004; Strommenger et al., 2008; Bosch et al., 2015; Darban-Sarokhalil et al., 2016; O'Hara et al., 2016). The spa gene contains three distinct regions: Fc, X, and C (Verwey, 1940; Harmsen et al., 2003; Goudarzi et al., 2016b). The polymorphic X region, which encodes a part of the staphylococcal protein A (Spa), contains variations in the number of tandem repeats and the base sequence within each repeat. In other words, each new sequence motif, with a length of 24 bp, found in any *S. aureus* strain is assigned a unique repeat code and the repeat succession and the precise sequences of the individual repeats for a given strain determines its *spa* type (Mazi et al., 2015). The primary binding site for protein A is the Fc region of mammalian immunoglobulins, most notably IgGs, which renders the bacteria inaccessible to opsonins, thus impairing phagocytosis by immune system attack (Graille et al., 2000).

According to the literature, the prevalence of *spa* types among *S. aureus* isolates varies in different areas around the world. According to the authors' knowledge, no comprehensive data, during the last decade, have been made available on the distribution of diverse *spa* types within different geographical areas, so the aim of the present study was to review the literature to report the most common clinical *spa* types which is important for discriminating *S. aureus* outbreak isolates and nosocomial infection control policies worldwide.

SeqNet.org has shown the 10 most frequent *spa* types on the seqNet during 2004–2008 which includes only the European countries plus Lebanon. These data seem to include MRSA from both human and veterinary sources which is different from the present review which includes only human clinical data and a larger geographic domain.

## Methods

### Search strategy and selection criteria

The PubMed, Google Scholar, Web of Science, Embase, Cochrane library, and Scopus databases were searched for original articles, reporting regionally prevalent *spa* types among *S. aureus* isolates. The search terms were “*Staphylococcus aureus, spa* typing.”

The articles were selected according to evaluations on titles, abstracts and the main text. The reasons for exclusion of certain articles were: non-human clinical isolates of *S. aureus*, old *spa*-typing methods (e.g., RFLP), non-English articles, and isolates from patients with certain specific diseases including HIV and other immune mediated afflictions. Following main text assessment, several articles were excluded for unspecified sample size or the lack of a predominant *spa* type in the study. Studies presented only in abstract form were also excluded. Papers occurring in more than a single database were cited once.

### Data extraction

The following data were extracted from each article: first author's name, year of publication, country, number of isolates, number of methicillin resistant and susceptible *S. aureus* isolates (MRSA and MSSA), the predominant *spa* type, SCCmec types of the predominant *spa* types, MLST and *spa* clonal complexes belonging to the most prevalent *spa* types.

## Results

**Figure 1:**
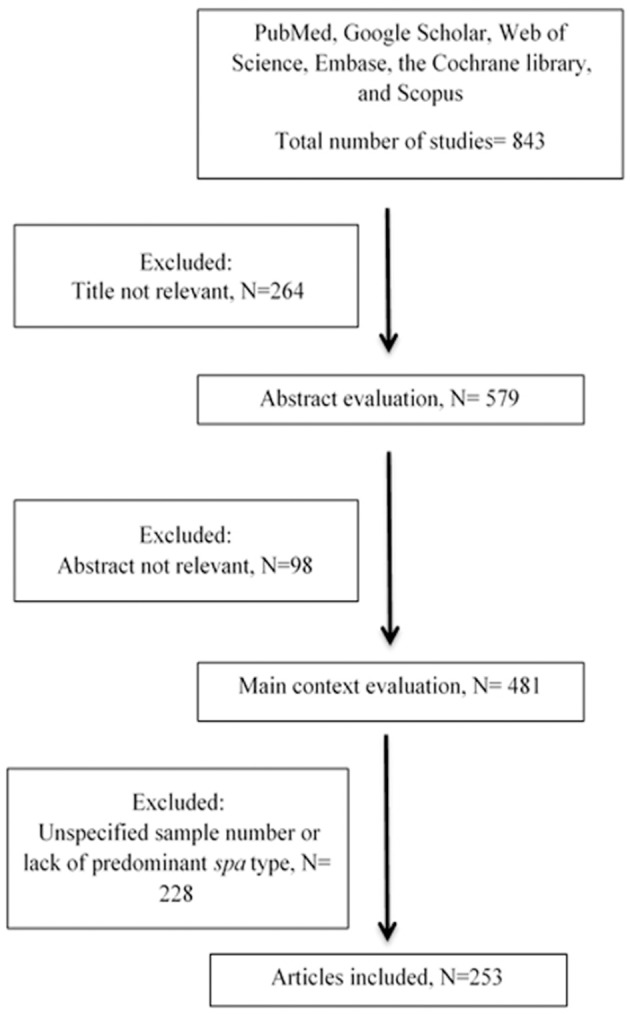
Flow diagram of literature search and study selection.

**Figure 2:**
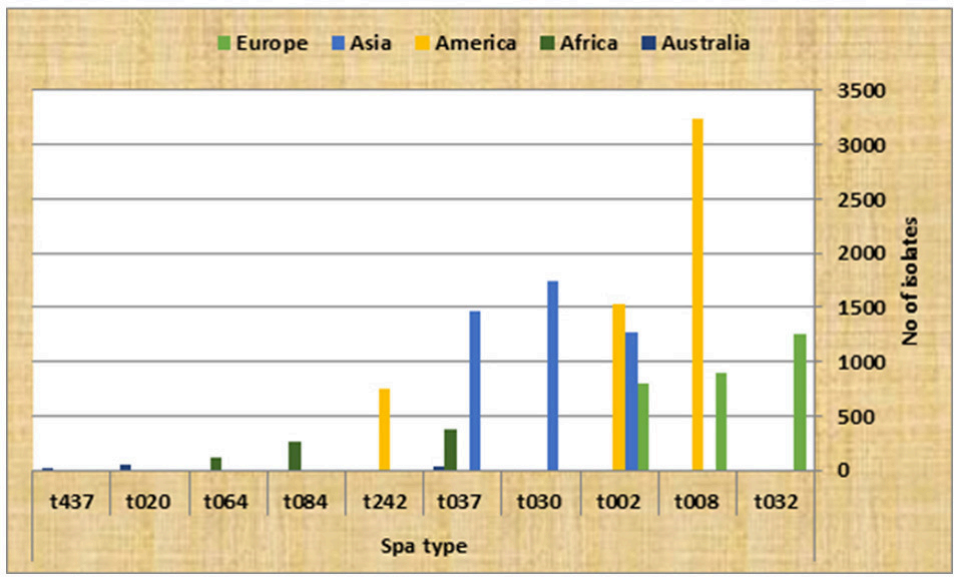
Three most prevalent *spa* types in Europe, Asia, America, Africa and Australia.

**Figure 3:**
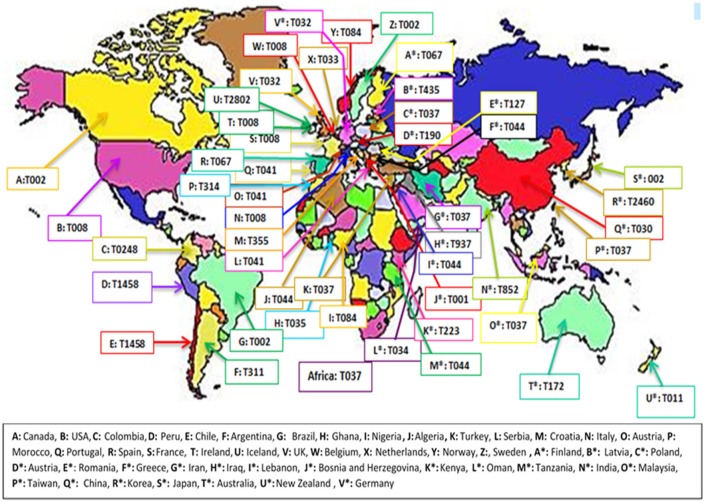
The most prevalent *spa* types across the world.

During the initial database search, a total of 843 articles, from 5 continents (Europe, Asia, America, Africa and Australia), were collected among which 264 and 98 were excluded based on title and abstract evaluations, respectively (Figure 1). Out of the remaining articles, 253 fulfilled our inclusion criteria (Shopsin et al., 1999; Graille et al., 2000; Fey et al., 2003; Arakere et al., 2005; Denis et al., 2005; Ko et al., 2005; Aires-de-Sousa et al., 2006, 2008; Deplano et al., 2006; Durand et al., 2006; Ferry et al., 2006, 2010; Fossum and Bukholm, 2006; Jury et al., 2006; Kuhn et al., 2006; Mellmann et al., 2006, 2008; Montesinos et al., 2006; Ruppitsch et al., 2006, 2007; Sabat et al., 2006; Cai et al., 2007, 2009; Conceicao et al., 2007; Cookson et al., 2007; Ellington et al., 2007a,b; Ghebremedhin et al., 2007; Hallin et al., 2007, 2008; Krasuski et al., 2007; Matussek et al., 2007; Otter et al., 2007; Tristan et al., 2007; Van Loo et al., 2007; Vourli et al., 2007; Werbick et al., 2007; Witte et al., 2007; von Eiff et al., 2007; Bartels et al., 2008, 2013, 2014; Chaberny et al., 2008; Chmelnitsky et al., 2008; Gardella et al., 2008; Fenner et al., 2008a,b; Golding et al., 2008, 2011; Ho et al., 2008a,b, 2012, 2016, 2017; Jappe et al., 2008; Karynski et al., 2008; Larsen et al., 2008, 2009; Nulens et al., 2008; Pérez-Vázquez et al., 2008; Strommenger et al., 2008; Vainio et al., 2008, 2011; Zhang et al., 2008, 2009; Alp et al., 2009; Argudín et al., 2009, 2011; Atkinson et al., 2009; Bekkhoucha et al., 2009; Chen et al., 2009; Chen H.-J. et al., 2010; Chen L. et al., 2010; Chen et al., 2012, 2013, 2014; Croes et al., 2009; Khandavilli et al., 2009; Köck et al., 2009; Lamaro-Cardoso et al., 2009; Lindqvist et al., 2009, 2012, 2015; Liu et al., 2009, 2010; Melin et al., 2009; Peck et al., 2009; Rasschaert et al., 2009; Rijnders et al., 2009; Shet et al., 2009; Soliman et al., 2009; Sun et al., 2009, 2013; Vindel et al., 2009; Argudin et al., 2010; Borghi et al., 2010; Coombs et al., 2010, 2012; Geng et al., 2010a,b,c; Ghaznavi-Rad et al., 2010; Graveland et al., 2010; Grundmann et al., 2010, 2014; Holzknecht et al., 2010; Ionescu et al., 2010; Laurent et al., 2010; Lee et al., 2010, 2013; Monaco et al., 2010; Moodley et al., 2010; Nadig et al., 2010, 2012; O'Sullivan et al., 2010; Petersson et al., 2010; Raulin et al., 2010; Ruimy et al., 2010; Shore et al., 2010, 2012, 2014; Valaperta et al., 2010; Wang et al., 2010, 2012, 2017; Alvarellos et al., 2011; Babouee et al., 2011; Blanco et al., 2011; Breurec et al., 2011a,b; Boakes et al., 2011; Cheng et al., 2011; Church et al., 2011; Conceição et al., 2011, 2012; García-Álvarez et al., 2011; Hesje et al., 2011; Jansen van Rensburg et al., 2011; Kechrid et al., 2011; Kim et al., 2011; Longtin et al., 2011; Miller et al., 2011; Skråmm et al., 2011; Pfingsten-Würzburg et al., 2011; Sanchini et al., 2011, 2014; Sangvik et al., 2011; Turlej et al., 2011; Ugolotti et al., 2011; Valentin-Domelier et al., 2011; Vandendriessche et al., 2011; Aamot et al., 2012, 2015; Adler et al., 2012; Berktold et al., 2012; Brennan et al., 2012; Cupane et al., 2012; Hafer et al., 2012; Hudson et al., 2012, 2013; Kriegeskorte et al., 2012; Lamand et al., 2012; Lim et al., 2012; Maeda et al., 2012; Marimón et al., 2012; Ngoa et al., 2012; Otokunefor et al., 2012; Ruffing et al., 2012; Sangal et al., 2012; Shambat et al., 2012; Sobral et al., 2012; Velasco et al., 2012; Blumental et al., 2013; Brauner et al., 2013; Camoez et al., 2013; Chroboczek et al., 2013a,b; David et al., 2013; Fernandez et al., 2013; García-Garrote et al., 2013; Gómez-Sanz et al., 2013; He et al., 2013; Japoni-Nejad et al., 2013; Kwak et al., 2013; Li et al., 2013; Lozano et al., 2013; Machuca et al., 2013; Medina et al., 2013; Miko et al., 2013; Murphy et al., 2013; Price et al., 2013; Prosperi et al., 2013; Sabri et al., 2013; Schmid et al., 2013; Song et al., 2013; Tian et al., 2013; Uzunović-Kamberović et al., 2013; van der Donk et al., 2013a,b; Williamson et al., 2013; Xiao et al., 2013; Aiken et al., 2014; Casey et al., 2014; Egyir et al., 2014; Faires et al., 2014; Harastani and Tokajian, 2014; Harastani et al., 2014; Havaei et al., 2014; Holmes et al., 2014; Kachrimanidou et al., 2014; Limbago et al., 2014; Luxner et al., 2014; Mohammadi et al., 2014; Rodríguez et al., 2014; Shakeri and Ghaemi, 2014; Tavares et al., 2014; Udo et al., 2014, 2016; Uzunović et al., 2014; Wiśniewska et al., 2014; Al Laham et al., 2015; Ayepola et al., 2015; Bartoloni et al., 2015; Biber et al., 2015; de Oliveira et al., 2015; Cirković et al., 2015,?; Mirzaii et al., 2015; O'Malley et al., 2015; Perovic et al., 2015; Rajan et al., 2015; Seidl et al., 2015; Shittu et al., 2015; Yu et al., 2015; Darban-Sarokhalil et al., 2016; Dündar et al., 2016; Garcia et al., 2016; Goudarzi et al., 2016a, 2017a,b; Jotić et al., 2016; O'Hara et al., 2016; Omuse et al., 2016; Parhizgari et al., 2016; Ahmed et al., 2017; Amissah et al., 2017; Bayat et al., 2017; Blomfeldt et al., 2017; Chmielarczyk et al., 2017; Gostev et al., 2017; Khemiri et al., 2017; Kong et al., 2017; Múnera et al., 2017; Pomorska-Wesołowska et al., 2017). In total, 127 articles were included from Europe, 70 from Asia, 33 from North and South America, 18 from Africa and 5 from Australia. More than 95% of the articles included in this study were published since 2007 and onwards. The frequent *spa* types on the different continents are shown in Figures 2, 3 and Table 1. The 3 most prevalent *spa* types were reported by 14, 33, and 22 out of the 127 studies in Europe, 13, 18, and 18 respective studies out of 70 in Asia, 13, 16, 2 out of 33 in America, and 3, 3, 4 out of 18 studies in Africa. Finally, in Australia, the 3 most prevalent *spa* types were reported by 1 article each out the total of 5 studies. In total, t202, t037, t437, t172, and t011 were the only *spa* types reported in Australia.

**Table 1:** Frequency of the common *spa* types among different continents.

**Continent**	**No. of isolates**				**The most predominant *spa* types (No. of isolates)**
	**MRSA**	**MSSA**	**Uncertified**	**Total**	
Europe	13,988	9,767	4,565	28,320	t032 (1,250), t008 (964), t002 (794), t044 (609), t003 (596), t067 (532), t018 (458), t004 (385)
Asia	6,903	1,383	329	8,615	t030 (2,009), t037 (1,591), t002 (1,277), t437 (351), t1081 (118), t004 (116), t001 (99), t2460 (65)
America	4,828	1,126	2,187	8,141	t008 (2,100), t002 (1,569), t242 (752), t012 (285), t084 (147), t003 (99), t311 (79), t0149 (74)
Africa	1,223	577	326	2,126	t037 (394), t084 (267), t064 (123), t1257 (120), t045 (79), t012 (68), t1443 (66), t314 (37)
Australia	148	44	0	192	t202 (50), t037 (32), t437 (19), t172 (8), t011 (6)

The Spa server has identified 17625 different *spa* types until the 17th of December, 2017^1^. Table 2 illustrates the distribution of diverse *spa* types among various SCC*mec* types in different continents. In Europe, 52 studies performed SCC*mec* typing on 3208 *spa* types and SCC*mec* types IV (1830 isolates) and II (800 isolates) were most associated and SCC*mec* type V (126 isolates) was least associated with the most common *spa* types. In Asia, SCC*mec* typing was performed on 4179 *spa* types by 41 studies and the most common *spa* types were classified into SCC*mec* types III (2725 isolates) and II (677 isolates), whilst the least number of *spa* types were categorized into SCC*mec* type V (104 isolates). A total of 12 studies in America performed SCC*mec* typing on 531 *spa* types, showing that SCC*mec* types IV (238 isolates) and II (167 isolates) were most associated with the frequent *spa* types. In Africa, 5 studies assessed the SCC*mec* types of 615 *spa* types and the common *spa* types were classified into SCC*mec* types IV (217 isolates) and V (185 isolates) whilst the least number of *spa* types were categorized into SCC*mec* type I (37 isolates). Finally, in Australia SCC*mec* typing was performed on 107 *spa* types by 5 studies and the most common *spa* types were classified into SCC*mec* types IV (49 isolates) and III (40 isolates), whilst the least number of *spa* types were categorized into SCC*mec* type I and II.

**Table 2:** Distribution of diverse *spa* types among various SCC*mec* types in different continents.

**Continent**	***Spa*** **types associated with SCC*****mec*** **types (No.)**				
	**Type I**	**Type II**	**Type III**	**Type IV**	**Type V**
Europe	t041 (189), t744 (8), t2023 (11), t002 (6), t022 (7), ?^*^ (71)	t018 (369), t003 (33), t002 (113), t004 (196), ?^*^ (89)	t037 (58), ?^*^ (102)	t032 (375), t008 (328), t067 (314), t019 (66), t2802 (37), t044 (164), t002 (49), t051 (10), t038 (7), t744 (8), t304 (31), t005 (32), t515 (18), t148 (14), t024 (42), t022 (12), t127 (61), t189 (4), t030 (11), ?^*^ (247)	t011 (60), t034 (31), t108 (11), t657 (23), t019 (1)
Asia	t127 (1), t2460 (1), t701 (2), t002 (8), t030 (3), t001 (99)	t002 (637), t2460 (36), t030 (4)	t037 (635), t071 (11), t030 (415), t002 (258), ?^*^ (1,406)	t852 (44), t190 (7), t127 (6), t002 (23), t324 (13), t008 (31), t437 (94), t796 (3), t318 (12), t991 (12), ?^*^ (203)	t701 (1), t002 (10), t030 (11), t081 (40), t437 (18), t657 (21), ?^*^ (3)
America	t149 (25), t149 (18)	t002 (44), t008 (3), ?^*^ (120)	t459 (29), t037 (7), ?^*^ (47)	t084 (135), t002 (3), t008 (38), t045 (3), t019 (23), t024 (25), t216 (6), ?^*^ (5)	–
Africa	t045 (37)	t311 (2), t012 (68)	t037 (106)	t044 (17), t311 (18), t186 (15), t064 (68), t1443 (66), t2196 (33)	t037 (1), t311 (1), ?^*^ (183)
Australia	–	–	t172 (8), t037 (32)	t437 (7), t202 (42)	t437 (12), t011 (6)

* *Unknown spa type*.

The total number of MRSA and MSSA isolates of the 3 most common *spa* types among different continents are shown in Table 3. In Europe, all the isolates related to *spa* type t032 were MRSA. In addition, *spa* type t037 in Africa and t037 and t437 in Australia were MRSA as well.

**Table 3:** Total number of MRSA and MSSA isolates of the most common *spa* types among different continents.

**Continent**	**The most common** ***spa*** **types (No.): No. of MRSA and/or MSSA isolates**		
Europe	t032 (1,250): 1250 MRSA	t008 (899): 510 MRSA, 229 MSSA, 90 uncertified	t002 (794): 450 MRSA, 100 MSSA, 244 uncertified
Asia	t030 (1,748): 1686 MRSA, 51 MSSA, 11uncertified	t037 (1,467): 1415 MRSA, 51 MSSA, 1 uncertified	T002 (1,285): 8064 MRSA, 9 MSSA, 340 uncertified
America	t008 (2,100): 2151 MRSA, 56 MSSA, 107 uncertified	t002 (1,525): 855 MRSA, 80 MSSA, 857 uncertified	t242 (752):478 MRSA, 274 uncertified
Africa	t037 (381): 381 MRSA	t084 (267): 217MSSA, 50 uncertified	t064 (123): 256 MRSA, 11 uncertified
Australia	t202 (50): 50 uncertified	t037 (32): 32 MRSA	t437 (19): 19 MRSA

*MRSA, methicillin resistant Staphylococcus aureus; MSSA, methicillin sensitive Staphylococcus aureus; Uncertified, not mentioned in the studies whether the isolates were MRSA or MSSA*.

*Spa* clonal complex (S-CC) and MLST clonal complex (M-CC), plus the sequence types (STs) of the most common *spa* types among different continents are illustrated in Table 4. The number of studies that reported *spa* clonal complex for the common *spa* types were 30 in Europe, 12 in Asia, 10 in America and 9 in Africa. Common *spa* types categorized into distinct MLST clonal complexes were 43 in Europe, 19 in Asia, 14 in America, and 9 in Africa. Forty eight studies in Europe, 29 in Asia, 18 in America, and 11 studies in Africa assessed the sequence types of the most common *spa* types. In Australia, no studies reported the *spa* or MLST complexes, nor any sequence types for the common *spa* types assessed.

**Table 4:** *Spa* and MLST clonal complexes plus sequence types of the most common *spa* types among different continents.

**Continent**	**Prevalent *spa* types (No. of isolates)**	***Spa* clonal complex/ S-CC (No. of *spa* types)**	**MLST clonal complex/M-CC (No. of *spa* types)**	**Sequence type/ST (No. of *spa* types)**
Europe	t032 (1,250) t008 (899) t002 (794)	– S-CC008 (22) S-CC002 (58)	M-CC22 (97) M-CC8 (57) M-CC5 (162)	ST22 (173) ST8 (295), ST247 (51) ST5 (186)
Asia	t030 (1,748) t037 (1,467) t002 (1,285)	S-CC030 (121) S-CC001 (111) S-CC002 (431), SCC001/002 (8)	M-CC59 (11), M-CC8 (159) M-CC8 (198), M-CC5 (8), M-CC 188 (16) M-CC5 (145), M-CC8 (157)	ST239 (1,422), ST22 (99) ST 239 (1,124) ST5 (459)
America	t008 (2,100) t002 (1,525) t242 (752)	S-CC008 (97) S-CC002 (53) –	M-CC 85 (85), M-CC5 (5) M-CC5 (30), M-CC8 (5) –	ST8 (524), ST247 (100) ST5 (701) –
Africa	t037 (381) t084 (267) t064 (123)	– S-CC84 (75) S-CC64 (68)	M-CC239 (30) M-CC15 (75) M-CC8 (10), M-CC30 (68)	ST 239 (173) ST 15 (60) ST8 (68)
Australia	t202 (50) t037 (32) t437 (19)	– – –	– – –	– – –

The association of the most prevalent *spa* types with different countries among different continents is shown by Table S1 in Supplementary Material. The data exhibit that The Netherlands has reported the most diverse range of *spa* types (34 types), followed by China (22 *spa* types), Germany (16 types), UK (15 types), Spain (11 types), Sweden and USA (10 *spa* types each), Italy and Iran (8 *spa* types each), France and Portugal (7 *spa* types each) and Switzerland (6 *spa* types).

Dissemination of different *spa* types among different countries is illustrated by Table S2 in Supplementary Material. The *spa* types t008 and t002 were the most frequently repeated *spa* types among the others, each repeated in 16 countries among different continent. The next most frequently repeated *spa* types were respectively t037 (12 countries), t044 (11 countries), t084 (8 countries), t012 and 127 (7 countries each), t041 (6 countries), and t019, t011, t034, t355, t189, t304 (5 countries each). Almost 50% of the *spa* types (43 out of the 87) were only reported by 1 country.

## Discussion

*Staphylococcus aureus* is capable of adapting to a variety of conditions and successful clones can be epidemic and even pandemic as can be concluded by their spreading from one continent to another (Parhizgari et al., 2016). The current review reports the prevalence of *spa* types among clinical isolates, both as carriage and infectious isolates, across the world. Our analysis showed that t032 was the most prevalent *spa* type in Europe, predominantly centered in the UK and Germany (Figure 3), and among the 5 most predominant *spa* types in Austria. No other countries in Europe have reported t032 among its most frequent *spa* types. Moreover, t008 was the second most prevalent and, nonetheless, the most frequently identified *spa* type in the various European countries, distributed among 11 out of the 22 of them investigating local *spa* types while also being the predominant type in France and Italy (Table S2 in Supplementary Material). Germany and UK principally provided a larger sample size compared to other European countries, looking over 10081 and 2644 isolates, respectively. Despite the fact that a larger sample size could be a proof to the validity of acquired data, it might also be that the disparity of the sample size among different countries has caused deviance in the report of the most prevalent *spa* type in Europe by the present study. Sweden appeared to be the only European country to have t002 as its most predominant *spa* type, even though t002 was disseminated in 9 out of the 22 European countries included in this analysis. A comprehensive molecular-epidemiological analysis, investigating the geographical distribution of invasive *S. aureus* isolates in Europe (Grundmann et al., 2010), revealed that the 3 most common *spa* types in Europe were t032, t008, and t002, respectively; which was in agreement with the results of the current meta-analysis. In Asia, t030 was the predominant *spa* type mainly located in China (Figure 3), while also reported by Iran as the fifth most common *spa* type. Moreover, t037, as the second most common *spa* type in Asia, was reported by more Asian countries compared to other *spa* types (Korea, China, Taiwan, Iran and Malaysia out of 10 Asian countries under this survey). Similarly in Africa, t037 was the most prevalent and t084 and t064 the most frequently repeated *spa* types, reported by 3 African countries each. Even though t008 was the most prevalent *spa* type in America, it was only reported by the USA and Canada. Then again, t002, as the second most common *spa* type was distributed among the USA, Canada and Brazil. Again, for these 3 continents the distinct sample size variation within the conforming countries might account for the different reports of the prevalent *spa* types among the associated continents. In Australia no precise information was revealed about the distribution of *spa* types.

The *spa* typing method, although being one of the valid schemes for the epidemiological surveillance of *S. aureus*, only considers a very limited portion of the whole genome and, therefore, could not possibly reflect the mutational events occurring in other parts of this organism's genome. Since certain *spa* types are still restricted to particular geographic locations, it might be considered that the polymorphic X region and, hence, the type of protein A have possible associations with the organism's adaptations to diverse conditions such as different host populations, the weather and geographical diversity.

As a vital virulence factor which enables the escape of *S. aureus* from innate and adaptive immune responses, the Spa protein may be an important target for adaptive evolution by means of host specialization and other environmental factors (Santos-Júnior et al., 2016). The plasticity of the *spa* gene, as a result of intragenic recombination, non-synonymous mutations as well as duplications events, can indeed influence the pathogenicity of *S. aureus*^1^. It has been shown that the mosaic *spa* gene is composed of different segments, each with a distinct evolutionary histories which could provide *S. aureus* with increased fitness to colonize the host surfaces or bind the immunoglobulin subunits. This diversity of Spa domains has contributed to the epidemic phenotype of *S. aureus* strains implying that they represent selected adaptations to their environment (Santos-Júnior et al., 2016).

Considering the fact that the primary binding site for protein A is the Fc region of mammalian immunoglobulins, and most notably IgGs (Graille et al., 2000), one possible justification for such an association might be the likely difference in the incidence rates of immunoglobulin subclasses among different geographical populations and, hence, the different binding strength of protein A types to these immunoglobulins. This might consequently cause a difference in the extent of opsonization and phagocytosis and, hence, the survival rates of particular *S. aureus spa* types within different populations (Sasso et al., 1991)^2^.

Overall, t008 (2692 MRSA, 258 MSSA, 222 uncertified) and t002 (9364 MRSA, 189 MSSA and 1441 uncertified) were the most widely distributed *spa* types worldwide, disseminated each through 16 out of the 34 countries assessing *spa* types, followed by t037 (1971 MRSA, 51 MSSA, 62 uncertified) and t044 (590MRSA, 0 MSSA, 77 uncertified) respectively occurring in 12 and 11 countries worldwide. Almost half of the *spa* types (43 out of the 87) were yet localized and limited to 1 country each (Table S2 in Supplementary Material). Migrations from one country/continent to another provides a reasonable justification as to why some *spa* types are common between certain countries/continents. In Europe, all the isolates related to *spa* type t032 were MRSA isolates. In addition, *spa* type t037 in Africa and t037and t437 in Australia consisted only of MRSA isolates; however, as shown in Table 3, the majority of predominant *spa* types consist of both MRSA and MSSA isolates (Adler et al., 2012; Jiménez et al., 2013; Aiken et al., 2014). Here again, a notable number of studies have not deduced whether the predominant *spa* types are MRSA or MSSA and there is therefore some missing points in the data regarding the association of prevalent *spa* types and methicillin resistance among different continents. Furthermore, results are dependent on the original sample collection to be *spa* typed. Most studies have, in the first place, *spa* typed methicillin resistant *S. aureus* isolates because of their epidemiological importance among clinical settings (Ruppitsch et al., 2006; Zhang et al., 2008; Miller et al., 2011) and therefore no specific conclusion is to be invoked as to whether MRSA/MSSA isolates belong to specific *spa* types or vice versa.

In Europe, SCC*mec* types IV and II were most associated with the common *spa* types. In Asia, the most common *spa* types were classified into SCC*mec* types III and II. In America, SCC*mec* types IV and II were most associated with frequent *spa* types. In Africa, the common *spa* types were classified into SCC*mec* types IV and V and finally, in Australia the most common *spa* types were classified into SCC*mec* types IV and III. The *spa* and SCC*mec* typing methods focus on two distinct locations within the genome of *S. aureus*. The last SCC*mec* type reported in 2015 in Germany was the SCC*mec* type XII (Wu et al., 2015), whereas the studies assessed in this review, have only ascertained limited SCC*mec* types (I, II, II, IV, and V). Moreover, a significant number of spa types have not been associated to any specific SCC*mec* type and the number of studies which have assessed SCC*mec* typing for the prevalent *spa* types are limited. For the above mentioned reasons, the association between certain *spa* and SCC*mec* types found in this review might be of questionable reliability.

Data relating to the *spa* and MLST clonal complexes, and sequence types of the most common *spa* types revealed that the spa clonal complexes (S-CC) 001 and 002 were common among Europe and Asia and had the highest association with prevalent *spa* types in this continents. Similarly, S-CC012 contained some frequent *spa* types reported by Europe, America and Africa while S-CC84 was only common among America and Africa. This means that some related *spa* types exist among different continents. On the other hand, MLST clonal complex (M-CC) 5 was associated with prevalent *spa* types in Europe, Asia, America and Africa. Meanwhile, some of the most frequently encountered *spa* types were associated to M-CC 8 which were common among Asia, America and Africa. It seems that there is a virtually sustained association between the *spa* and sequence types irrelevant of the continent. For example, t032 has almost always been associated with ST22 across all continents; the same is true for t008 which has been associated with either ST8 or ST247, among all the studies being assessed in this review. As some of the most prevalent *spa* types reported by many different studies, t002, t030, and t037 have been constantly associated with ST5; ST239 and ST22; and ST239, respectively. In Australia, no studies reported the *spa*, MLST complexes or sequence types for the common *spa* types assessed. *S. aureus*, as an organism with a relatively stable genome, tends to present as clones which are relatively stable and generally diversify by the accumulation of single nucleotide substitutions without frequent inter-strain recombination (Grundmann et al., 2010; Shittu et al., 2011; van der Donk et al., 2013b). It is also noteworthy to mention that *S. aureus* clones might vary among different clinical settings within the same country or even among different wards of the same hospital (Shittu et al., 2011; van der Donk et al., 2013b; Seidl et al., 2015). Since a majority of studies under this review did not specifically discern the exact location of sampling, the data presented in this review presents a general information about the prevalent *spa* types and the associated clonal complexes in each country/continent; so, it would have been valuable to provide information on the exact sampling time and location within each country among different continents.

## Conclusion

This review shows the spread of the most prevalent *spa* types in countries, continents and worldwide. Such data can be used for epidemiological purposes, such as defining the geographical spread of the predominant *spa* types of *S. aureus*, the interpretation of relative frequencies, comparing the worldwide diverse evolutionary trajectories of *S. aureus* lineages, and the understanding of molecular epidemiological dynamics of *S. aureus* transmission.

## Author contributions

DD-S designed the first concept, helped in the literature review, data extraction, and preparation of the manuscript. PA prepared the manuscript, interpreted the data, and helped in the literature review and data extraction. NF helped in the literature review and data extraction. MM, SSK, and MD participated in the manuscript preparation. AvB made critical revision and helped in the preparation of the manuscript. KA helped in the analysis, interpretation of data, and preparation of the manuscript. All authors read and confirmed the content of the paper.

### Conflict of interest statement

The authors declare that the research was conducted in the absence of any commercial or financial relationships that could be construed as a potential conflict of interest.

## Footnotes

^1^Ridom Gmb, H., info@ridom.de, Rothgaenger, J., Harmsen, D. Molecular diagnostic differentiation and typing of bacteria - software solutions. (8601); published online Epub(SCHEME = ISO8601) 2003-11-02 (http://www.ridom.de/).

^2^G. healthcare. (http://www.gelifesciences.com), pp. DF-1.6 %âãÏÓ 1333 1330 obj <</Linearized 1331/L 3256349/O 3251336/E 3272957/N 3256173/T 3254047/H [3256516 3251548]>> endobj 3251354 3256340 obj <</DecodeParms <</Columns 3256344/Predictor 3256312>>/Filter/ FlateDecode/ID[ <3256387AC3256329D3256370FC3256363F3256344D3256 345CCEBFB3256374F3256335F3256811> <3256819D3256377F3256347 F3829348BEF3256342C3256343C3256931C3256359C3256346F>]/Index[325 1333 3256336]/Info 3251332 3256340 R/Length 3256100/Prev 3254048/Root 3251334 3256340 R/Size 3251369/Type/XRef/W[3256341 3256342 3256341]>>stream hÞbbd “bà”.
