# Supplementary material for: Distribution of the Most Prevalent Spa Types among Clinical Isolates of Methicillin-Resistant and -Susceptible Staphylococcus aureus around the World: A Review
Source: Front Microbiol. 2018 Feb 12;9:163. doi: 10.3389/fmicb.2018.00163 (PMC5816571; doi:10.3389/fmicb.2018.00163)
Supplement: Supplementary file 1 [file Table1.docx]

| **Table S1.** Association of the prevalent *spa* types with different countries among different continents | | |
| --- | --- | --- |
| **Continent** | **Country (No. isolates)** | **Number of prevalent *spa* types** |
| **Europe** | **Finland (757)** | 159 t067,95 t172, 5 t044 |
|  | **Norway (2535)** | 176 t084, 122 t012,96 t657, 55 t065, 54 t044, 12 t067 |
|  | **UK (2644)** | 456 t032, 411 t018, 345 t044, 77 t127, 73 t037, 62 t008, 44 t190, 39 t657, 35 t005, 22 t019, 12 t022, 11 t025, 8 t002, 6 t023, 6 t012 |
|  | **France (1079)** | 165 t008, 49 t571, 44 t002, 31 t304, 18 t011, 12 t024, 11 t376 |
|  | **Spain (1243)** | 349 t067, 196 t004, 58 t008, 50 t018, 21 t011, 20 t002, 14 t148, 11 t012, 6 t034, 6 t651, 3 t163 |
|  | **Poland (286)** | 54 t003, 46 t037, 11 t053, 7 t127, 7 t021 |
|  | **Belgium (660)** | 27 t008, 9 t044, 8 t744 |
|  | **Russia (480)** | 65 t008 |
|  | **Austria (972)** | 211 t190, 63 t032, 58 t008, 36 t041, 11 t202, 8 t009 |
|  | **Netherlands (2558)** | 276 t033, 202 t002, 202 t019, 216 t108, 188 t515, 181 t230, 162 t172, 153 t744, 133 t128, 124 t922, 123 t008, 141 t728, 116 t435, 107 t004, 107 t127, 99 t003, 88 t355, 87 t843, 87 t376, 87 t190, 63 t189, 53 t091, 51 t005, 38 t034, 15 t037, 15 t701, 14 t433, 10 t053, 8 t011, 8 t021, 8 t073, 8 t075, 8 t163, 8 t078 |
|  | **Sweden (851)** | 88 t002, 51 t149, 31 t044, 30 t012, 24 t355, 15 t084, 10 t015, 5 t019, 5 t051, 12 t067 |
|  | **Germany (10081)** | 631 t032, 418 t003, 162 t004, 100 t008, 93 t002, 68 t001, 39 t011, 39 t005, 31 t034, 25 t108, 11 t843, 11 t336, 7 t318, 6 t020, 5 t311, 5 t022 |
|  | **Serbia (30)** | 8 t041, 7 t022 |
|  | **Iceland (226)** | 37 t280, 28 t019 |
|  | **Switzerland (601)** | 51 t041, 48 t002, 41 t008, 15 t020, 13 t127, 8 t084 |
|  | **Italy (484)** | 114 t008, 56 t002, 31 t041, 18 t515, 9 t001, 5 t044, 4 t189, 4 t701 |
|  | **Croatia (23)** | 19 t355 |
|  | **Greece (55)** | 26 t044, 11 t003, 5 t037 |
|  | **Romania (296)** | 39 t127, 32 t351, 11 t030, 7 t008, 7 t044 |
|  | **Portugal (824)** | 141 t041, 136 t002, 50 t012, 22 t008, 19 t159, 7 t1228, 6 t318 |
|  | **Turkey (270)** | 219 t030, 48 t037 |
|  | **Bosnia and Herzegovina (147)** | 16 t001, 14 t003, 11t041, 10 t005, 9 t728 |
|  | **Riga Latvia (224)** | 52 t435 |
| **Asia** | **Korea (256)** | 28 t246, 24 t002, 6 t189, 23 t037, 25 t190, 18 t664, 13 t324 |
|  | **China (6013)** | 1737 t030, 959 t037, 943 t002, 400 t081, 203 t437, 105 t1081, 60t311, 37 t2460, 36 t189, 31 t3297, 32 t084, 27 t571, 17 t127, 14 t309, 13 t081, 13 t338, 12 t034, 12 t318, 11 t4677, 11 t091, 11 t045, 10 t796 |
|  | **India (110)** | 56 t852, 21 t657 |
|  | **Taiwan (683)** | 240 t037,184 t002, 148 t437, 13 t189 |
|  | **Lebanon (299)** | 116 t044, 36 t223, 19 t021 |
|  | **Iran (910)** | 448 t037, 56 t790, 53 t030, 30 t761, 29 t937, 28 t631, 25 t1149, 19 t969, 13 t7688, 9 t267, 4 t304 |
|  | **Japan (108)** | 67 t002, 5 t008 |
|  | **Oman (79)** | 7 t034, 7 t304 |
|  | **Malaysia (221)** | 228 t037, 12 t177, 21 t421 |
|  | **Kuwait (37)** | 19 t223 |
|  | **Palastine/Iraq/Jordan (12)** | 11 t937 |
| **America** | **Canada (1279)** | 148 t002, 19 t001, 12 t008, 9 t128 |
|  | **USA (5742)** | 1965 t008, 838 t002, 752 t242, 147 t084, 46 t311, 29 t459, 23 t065, 12 t012, 9 t064, 6 t216 |
|  | **Colombia (990)** | 41 t1610, 28 t024, 25 t149, 3 t045 |
|  | **Argentina (97)** | 33 t311, 23 t019, 10 t021 |
|  | **Brazil (46)** | 13 t002, 7 t037 |
|  | **Chile (29)** | 25 t145 |
|  | **Peru (64)** | 24 t148, 18 t149 |
| **Africa** | **Western Algeria (61)** | 17 t044, 15 t223, 11 t008 |
|  | **Morocco (246)** | 37 t314, 32 t127, 25 t084 |
|  | **Ghana (124)** | 18 t355, 14 t008, 13 t10519, 21 t928 |
|  | **Kenya (86)** | 15 t223, 10 t064, 13 t037 |
|  | **Nigeria (328)** | 192 t084, 13 t230, 12 t843, 11 t064, 10 t355 |
|  | **Tanzania (24)** | 8 t044, 7 t690, 7 t7231 |
|  | **Libya (32)** | 32 t6065 |
|  | **Uncertified (1221)** | 381 t037, 120 t1257, 102 t064, 79 t045, 68 t012, 66 t1443, 50 t084, 33 t2196 , 21 t311, 15 t186 |
| **Australia** | **New Zealand (9)** | 6 t011 |
|  | **Uncertified (183)** | 80 t172, 50 t202, 32 t037, 19 t437 |
