# Supplementary material for: Distribution of the Most Prevalent Spa Types among Clinical Isolates of Methicillin-Resistant and -Susceptible Staphylococcus aureus around the World: A Review
Source: Front Microbiol. 2018 Feb 12;9:163. doi: 10.3389/fmicb.2018.00163 (PMC5816571; doi:10.3389/fmicb.2018.00163)
Supplement: Supplementary file 2 [file Table2.docx]

| **Table S2.** Dissemination of different *spa* types among different countries | | | |
| --- | --- | --- | --- |
| ***Spa* type** | **Country** | ***Spa* type** | **Country** |
| t008 | UK, France, Spain, Belgium, Austria, Netherland, Germany, Switzerland, Italy, Malaysia, Portugal, Japan, Canada, USA, Algeria, Ghana, Russia928 | t246 | Korea, China |
| t002 | UK, France, Spain, Netherland, Sweden, Germany, Switzerland, Italy, Portugal, Korea, China, Taiwan, Japan, Canada, USA, Brazil | t937 | Iran, Iraq |
| t037 | UK, Poland, Netherland, Greece, Turkey, Korea, China, Taiwan, Iran, Malaysia, Brazil, Australia, Kenya | t025 | UK |
| t044 | Finland, Norway, UK, Belgium, Sweden, Italy, Greece, Romania, Lebanon, Algeria, Tanzania | t023 | UK |
| t084 | Norway, Sweden, Switzerland, China, USA, Morocco, Africa, Nigeria | t651 | Spain |
| t012 | Norway, UK, Spain, Sweden, Portugal, USA, Africa | t163 | Spain |
| t127 | UK, Poland, Netherland, Switzerland, Romania, China, Morocco | t033 | Netherland |
| t041 | Austria, Serbia, Switzerland, Italy, Portugal, Bosnia and Herzegovina | t009 | Austria |
| t019 | UK, Netherland, Sweden, Iceland, Argentina | t922 | Netherland |
| t011 | France, Spain, Netherland, Germany, Australia | t433 | Netherland |
| t034 | Spain, Netherland, Germany, China, Oman | t073 | Netherland |
| t355 | Netherland, Sweden, Croatia, Ghana, Nigeria | t075 | Netherland |
| t189 | Netherland, Italy, Korea, China, Taiwan | t078 | Netherland |
| t304 | Spain, Netherland, Germany, China, Oman | t015 | Sweden |
| t190 | UK, Austria, Netherland, Korea | t051 | Sweden |
| t005 | UK, Netherland, Germany, Bosnia and Herzegovina | t336 | Germany |
| t021 | Netherland, Poland, Lebanon, Argentina | t280 | Iceland |
| t003 | Poland, Germany, Greece, Bosnia and Herzegovina | t351 | Romania |
| t001 | Germany, Italy, Bosnia and Herzegovina, Canada | t159 | Portugal |
| t311 | Germany, USA, Argentina, Africa | t318 | Portugal |
| t064 | USA, Africa, Kenya, Nigeria | t728 | Bosnia and Herzegovina |
| t067 | Finland, Norway, Spain, Sweden | t324 | Korea |
| t172 | Finland, Netherland, Australia | t664 | Korea |
| t032 | UK, Germany, Austria | t1228 | Portugal |
| t022 | UK, Germany, Serbia | t1081 | China |
| t004 | Spain, Netherland, Germany | t2460 | China |
| t108 | Netherland, Germany, China | t3297 | China |
| t843 | Netherland, Germany, Africa | t309 | China |
| t318 | Germany, Portugal, China | t338 | China |
| t030 | Romania, China, Iran | t4677 | China |
| t437 | China, Taiwan, Australia | t796 | China |
| t223 | Lebanon, Algeria, Kenya, Kuwait | t852 | Netherland |
| t065 | Norway, USA | t189 | Taiwan |
| t018 | UK, *Spa*in | t761 | Iran |
| t657 | UK, India, Norway | t7688 | Iran |
| t024 | France, Colombia | t267 | Iran |
| t0571 | France, China | t709 | Iran |
| t376 | France, Netherlands | t177 | Malaysia |
| t148 | Spain, Peru | t421 | Malaysia |
| t053 | Poland, Netherland | t242 | USA |
| t744 | Belgium, Poland | t459 | USA |
| t202 | Austria, Australia | t216 | USA |
| t230 | Netherland, Nigeria | t145 | Chile |
| t172 | Netherland, Australia | t314 | Morocco |
| t515 | Netherland, Australia | t1257 | Africa |
| t128 | Netherland, Canada | t1443 | Africa |
| t728 | Netherland, Bosnia and Herzegovina | t2196 | Africa |
| t435 | Netherland, Riga Latvia | t189 | Africa |
| t091 | Netherland, China | t10519 | Ghana |
| t701 | Netherland, Italy | t690 | Tanzania |
| t149 | Netherland, Peru | t7231 | Tanzania |
| t020 | Germany, Switzerland | t928 | Ghana |
| t6065 | Libya | t790 | Iran |
| t1149 | Iran | t969 | Iran |
| t631 | Iran | t1610 | Colombia |
